# Lytic polysaccharide monooxygenases: enzymes for controlled and site-specific Fenton-like chemistry

**DOI:** 10.1042/EBC20220250

**Published:** 2023-04-18

**Authors:** Bastien Bissaro, Vincent G.H. Eijsink

**Affiliations:** 1 INRAE, Aix Marseille University, UMR1163 Biodiversité et Biotechnologie Fongiques, 13009 Marseille, France; 2 Faculty of Chemistry, Biotechnology, and Food Science, The Norwegian University of Life Sciences (NMBU), 1432 Ås, Norway

**Keywords:** copper, Fenton, LPMO, peroxygenase, polysaccharides

## Abstract

The discovery of oxidative cleavage of glycosidic bonds by enzymes currently known as lytic polysaccharide monooxygenases (LPMOs) has profoundly changed our current understanding of enzymatic processes underlying the conversion of polysaccharides in the biosphere. LPMOs are truly unique enzymes, harboring a single copper atom in a solvent-exposed active site, allowing them to oxidize C-H bonds at the C1 and/or C4 carbon of glycosidic linkages found in recalcitrant, often crystalline polysaccharides such as cellulose and chitin. To catalyze this challenging reaction, LPMOs harness and control a powerful oxidative reaction that involves Fenton-like chemistry. In this essay, we first draw a brief portrait of the LPMO field, notably explaining the shift from the monooxygenase paradigm (i.e., using O_2_ as cosubstrate) to that of a peroxygenase (i.e., using H_2_O_2_). Then, we briefly review current understanding of how LPMOs generate and control a hydroxyl radical (HO^•^) generated through Cu(I)-catalyzed H_2_O_2_ homolysis, and how this radical is used to create the proposed Cu(II)-oxyl species, abstracting hydrogen atom of the C-H bond. We also point at the complexity of analyzing redox reactions involving reactive oxygen species and address potential deficiencies in the interpretation of existing LPMO data. Being the first copper enzymes shown to enable site-specific Fenton-like chemistry, and maybe not the only ones, LPMOs may serve as a blueprint for future research on monocopper peroxygenases.

## A bit of history

In 1974, Eriksson et al. showed that cellulose degradation by a fungal secretome was more efficient in the presence of oxygen. At the time, this finding directed attention to an enzyme called cellobiose dehydrogenase (initially called cellobiose oxidase [1]). It was not until 2010 that it was shown that bacterial and fungal secretomes contain enzymes, today referred to as lytic polysaccharide monooxygenases (LPMOs), which oxidize polysaccharides (such as chitin and cellulose) in an oxygen-dependent manner and that, by doing so, increase the efficiency of classical hydrolases. The existence of these enzymes was first revealed by studying chitin degradation [2]. First glimpses of what was coming appeared in 2005, when Vaaje-Kolstad et al. showed that a protein, at the time thought to be a family 33 carbohydrate-binding module (CBM33), boosts the activity of chitinases [3,4], and in 2007/2008, when Merino & Cherry and Karkehabadi et al. showed that proteins, at the time thought to be a family 61 glycoside hydrolases (GH61), are structurally similar to CBM33s and boost the activity of cellulases [5,6].

In the past decade, LPMOs have gained a lot of attention, because of their intriguing catalytic ability and mechanism, their documented value as a key component of enzyme cocktails for industrial processing of lignocellulosic biomass [7–10], and the emerging notion that these taxonomically widespread and abundant enzymes may be involved in other biological processes [11], such as cellular development [12,13] and virulence [14–16]. Today, LPMOs are classified as auxiliary activities (AA) in the carbohydrate-active enzymes (CAZy) database and distributed in eight families, namely AA9 to AA11 and AA13 to AA17. LPMOs have the unique ability to act on solid–liquid interfaces, meaning that they can degrade polysaccharides that are embedded in an insoluble, sometimes even crystalline structure [17,18]. This is very different from classical hydrolytic enzymes, such as cellulases, which act on single ‘decrystallized’ polysaccharide chains [2]. LPMOs have evolved to overcome the exceedingly high energy barrier (approximately 95–104 kcal/mol; [19–21]) associated with C-H bond activation in the glycosidic bonds of crystalline polysaccharides. The chemistry afforded by combining and orienting the 20 naturally occurring amino acids does not suffice to overcome this barrier; hence, LPMOs have evolved an active site equipped for controlled metal-catalyzed generation of a highly oxidative oxygen species.

While the first crystallographic studies of proteins known today as LPMOs showed the presence of a single metal-binding site [3,6], the nature of this metal ion remained unclear [2,22], until it was established in 2011 [23,24] that LPMOs are monocopper enzymes. The copper is bound by a highly conserved arrangement of two histidine residues referred to as the histidine-brace [24–27]. Although the catalytic mechanism of LPMOs remains unknown in part (see below), it is generally accepted that LPMO action entails the formation of a powerful reactive oxygen species on the copper ion, possibly a copper(II)-oxyl ([CuO]^+^) [19,28–30]. In cellulose-active LPMOs, this oxygen species abstracts a hydrogen atom from the C1 or the C4 of the scissile glycosidic bond, followed by substrate hydroxylation through an oxygen-rebound mechanism. Hydroxylation destabilizes the glycosidic bond, leading to cleavage and formation of one normal and one oxidized new chain end [23,31].

Regardless of the fine mechanistic details underlying the formation of the copper(II)-oxyl species, oxygen atoms must be recruited at some point during catalysis. In the first study showing that LPMOs catalyze oxidative cleavage of polysaccharides, experiments with ^18^O_2_ showed incorporation of one ^18^O in the oxidized products [2]. Thus, when the nature of the bound metal ion was found to be copper [23,24,32], by analogy to enzymes such as particulate methane monooxygenase and ammonia monooxygenase, the logical conclusion was that LPMOs are monooxygenases. The monooxygenase reaction requires two externally delivered electrons and one O_2_ molecule to catalyze the following reaction: R-H + O_2_ + 2H^+^ +2e^−^ → R-OH + H_2_O. Accordingly, typical set-ups for LPMO reactions entail mixing the enzyme with a substrate and an electron source, under aerobic conditions. While ascorbic acid is commonly used as electron source, LPMO reactions can be fueled by a wide range of reductants as well as by enzymes capable of delivering electrons, such as cellobiose dehydrogenase [33–35].

The LPMO copper site is remarkably solvent-exposed and its reactivity will be heavily affected by the presence of substrate, which will shield the copper from solvent, displace water molecules, and change the electronic environment of the metal ion. Such effects have indeed been revealed by both crystallographic studies [36,37], modeling [38], and EPR spectroscopy [38–40]. Importantly, when not bound to substrate, LPMOs may, upon reduction, engage in off-pathway reactions. First, reduced LPMOs may react with molecular oxygen to generate hydrogen peroxide [41] in what is referred to as an oxidase reaction. Reduced LPMOs may also react with hydrogen peroxide in the reaction solution and this may lead to oxidative damage to the LPMO active site (see below). The occurrence of such off-pathway reactions will depend on whether or not an appropriate substrate is present, and on the substrate concentration.

**Figure 1:**
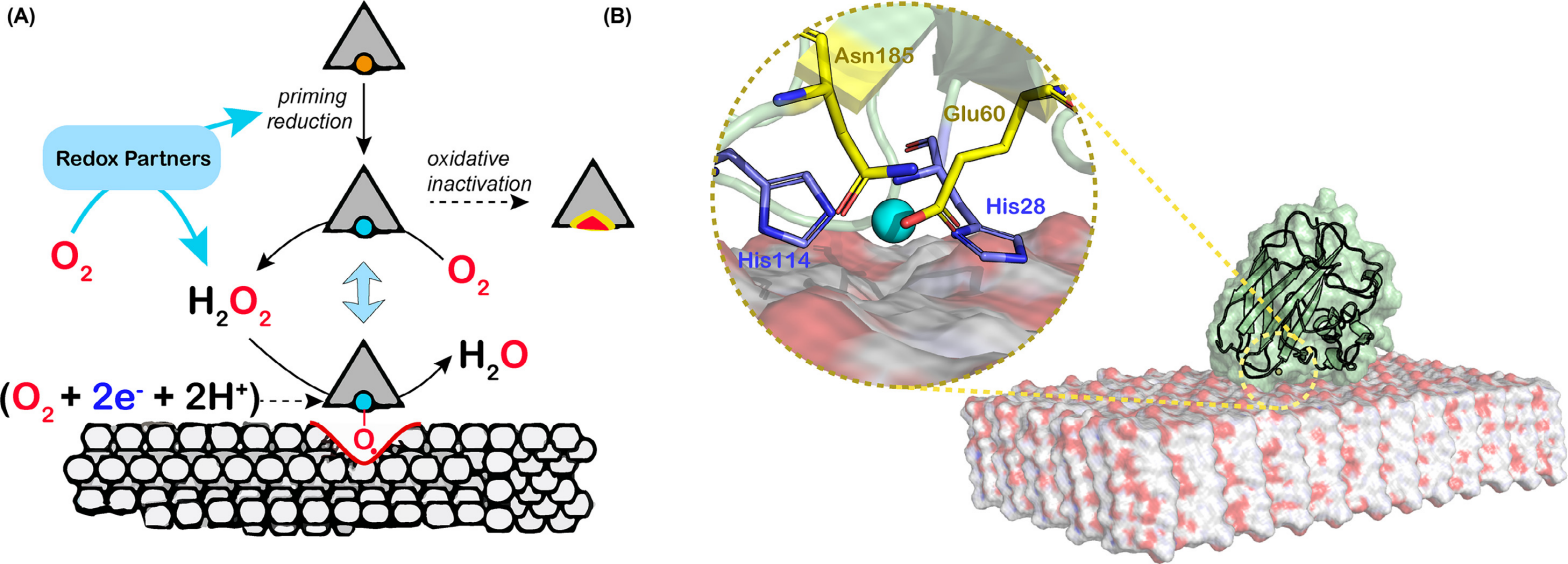
Schematic view of the peroxygenase action of LPMOs on a crystalline polysaccharide (**A**) The LPMO-Cu(II) form (copper atom shown as orange sphere) is reduced to the Cu(I) form (blue sphere) by a redox partner (small organic reductant, redox protein, photoactivated molecule). LPMO-Cu(I) molecules remaining in the unbound state (e.g. because of lack of a genuine substrate) can catalyze an oxidase reaction (production of H_2_O_2_) and are susceptible to oxidative inactivation. LPMO-Cu(I) molecules bound to the polysaccharide will catalyze a peroxygenase reaction leading to chain cleavage. The alternative, hypothetical monooxygenase reaction is shown in between parenthesis and entails the delivery of two electrons and two protons per catalytic cycle. (**B**) Model of an LPMO (the AA10 from *Serratia marcescens*) bound to β-chitin [38]. The inset shows the entrance to a tunnel that connects the active site to the bulk solvent, including the residues Glu60 and Asn185 (shown as yellow sticks) that limit tunnel accessibility to small molecules only (such as O_2_, H_2_O_2_, or H_2_O). The histidine brace is shown as blue sticks.

Intriguingly, in contrast with other copper monooxygenases, LPMOs contain only one copper ion and can thus store only one of the two electrons needed in a monooxygenase reaction. The path by which the second electron reaches the catalytic center is not immediately obvious since, during catalysis, the LPMO is secluded from the solvent by the polymeric, insoluble substrate (Figure 1). Several researchers have proposed the existence of electron-transport paths in LPMOs, but the enzymes do not show conserved structural features that might relate to such paths. This issue has sometimes been referred to as the ‘second electron conundrum’.

Although perhaps somewhat less visible in the literature, kinetic data obtained using standard ‘monooxygenase reaction conditions’ raise additional intriguing issues. First, reported progress curves for LPMO reactions tend to be nonlinear, which reflects that LPMO reactions are hard to control, with possible reductant depletion and/or enzyme inactivation. Second, the reported ‘monooxygenase’ rates tend to be excessively low, usually below 1 per min (see ref. [42], for an overview).

Intrigued by these observations and considerations, and inspired by a study on light-driven LPMO catalysis showing unprecedented high catalytic rates [43], we questioned established views on LPMO catalysis and proposed, in 2016, based on a large series of experiments [44,45], that LPMOs are in fact peroxygenases, i.e. using hydrogen peroxide rather than molecular oxygen as the cosubstrate (Figure 1). Originally, our findings and proposal were presented under the title ‘Fenton-type chemistry by a copper enzyme: molecular mechanism of polysaccharide oxidative cleavage’ [44]. This proposal was controversial [46,47] because it entailed the existence of a new type of peroxygenase catalysis not requiring any other cofactor than a single copper (rather than a heme). At the same time, this proposal was attractive because it was, and still is, compatible with existing data and provided reasonable explanations for outstanding issues:

- Apparent monooxygenase activity may very well be peroxygenase activity. In all typical LPMO reaction set-ups, H_2_O_2_ will be formed *in situ*, through (i) oxidation of the reductant (either LPMO-catalyzed—i.e. via the oxidase activity—or via abiotic, free metal-catalyzed oxidation of the reductant; [48,49]) and/or (ii) the possible oxidase activity of an enzymatic redox partner such as cellobiose dehydrogenase [50,51]. This also applies to the experiment with ^18^O_2_ conducted in 2010, where H_2_^18^O_2_ was likely formed *in situ*. Thus, in these typical ‘monooxygenase’ reaction set-ups, the peroxygenase reaction can take place.
- The ‘2^nd^ electron conundrum’. A peroxygenase reaction (R-H + H_2_O_2_ →R-OH + H_2_O) only requires a priming reduction of the LPMO and does not require delivery of electrons during catalysis (Figure 1).
- The excessively low catalytic rate of LPMOs. Both our original work [44,45] and follow-up studies [52–60] have shown that the peroxygenase reaction is orders of magnitude faster than the (apparent) monooxygenase reaction and that under ‘peroxygenase conditions’ kinetic parameters for LPMOs resemble those of other peroxygenases [52,54]. Today, it is clear that under standard ‘monooxygenase’ conditions (i.e. presence of a reductant, such as ascorbic acid, under aerobic conditions, with no added H_2_O_2_), the LPMO reaction rate is limited by the usually very slow *in-situ* generation of H_2_O_2_.
- The instability of LPMOs. Reactions with ‘too much’ H_2_O_2_ lead to oxidative damage of the LPMO active site, showing that the regularly observed inactivation of LPMOs under turnover conditions is due to an autocatalytic process [45]. It is now generally accepted that reduced LPMOs that meet H_2_O_2_ in the absence of substrate catalyze a slow peroxidase reaction that may lead to damage [61]. Such damage, either caused by too much added H_2_O_2_ or by too high levels of *in-situ* generated H_2_O_2_, explains why LPMO reactions tend to show nonlinear progress curves and signs of enzyme inactivation. This may also explain why Scott et al. observed that addition of catalase, which will help keeping H_2_O_2_ levels low, improved the performance of an LPMO-containing cellulase cocktail in saccharification of lignocellulosic biomass [62].

Today, it is clear that LPMOs are efficient peroxygenases and most researchers would believe that the activity and stability of these enzymes, in laboratory reactions and industrial bioprocessing plants alike, depend on access to H_2_O_2_.

## LPMOs are truly special: controlled, site-specific Fenton-like chemistry

**Figure 2:**
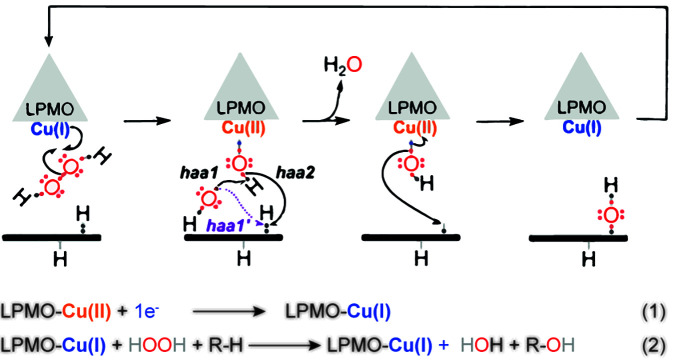
Reaction scheme proposed when the LPMO peroxygenase reaction was first described LPMO-Cu(II) is first reduced to LPMO-Cu(I) (‘priming reduction’), followed by H_2_O_2_ binding and homolytic bond cleavage. This cleavage leads to the Fenton-like generation of a hydroxyl radical, catalyzing haa either from the Cu(II)-hydroxide (haa1) or from the substrate (haa1’). The former scenario generates a Cu(II)-oxyl intermediate that can abstract a hydrogen atom from the substrate (haa2). Later studies have shown that haa1 (followed by haa2), rather than haa1’, is the most plausible scenario. The formation of the Cu(II)-hydroxide/substrate radical complex is followed by hydroxylation of the substrate via an oxygen-rebound mechanism and concomitant regeneration of the Cu(I) center. This picture and most of the legend were taken from Figure 2 in [44].

At the time when LPMOs were still considered to be monooxygenases, it had been anticipated, based on calculations and chemical intuition, that the reactive species capable of abstracting a hydrogen atom from the C1 or C4 in crystalline cellulose would need to be a Cu(II)-oxyl ([CuO]^+^) or perhaps its protonated form, Cu(III)-hydroxo ([CuOH]^2+^) [28,63–65] (see Figure 2). While it had been shown that reduced LPMOs react with molecular oxygen [66], the resulting Cu(II)-superoxo ([CuO_2_]^+^) species was not considered sufficiently powerful to abstract the hydrogen atom [19,28]. Interestingly, almost all later calculations, considering a monooxygenase reaction, a peroxygenase reaction, or both, predicted Cu(II)-oxyl as the most likely species catalyzing hydrogen atom abstraction (haa), the rate-limiting step during substrate oxidation [19,29,67,68].

Several possible catalytic scenarios, all converging toward a common point, i.e. a Cu(II)-hydroxide/substrate radical complex (Figure 2, third state), were considered when the peroxygenase activity of LPMOs was first described (see the Supplementary Materials of [44]). These scenarios involve either heterolytic or homolytic cleavage of the H_2_O_2_ molecule and the latter scenario was considered most plausible [44] (Figure 2). Subsequent studies by multiple groups have concluded that, indeed, homolytic cleavage occurs, leading to the formation of a Cu(II)-hydroxide and a hydroxyl radical (HO^•^) [19,29,67,68]. Such homolytic cleavage has been experimentally evidenced by the detection of hydroxyl radicals in work by Bissaro et al. [68] and Jones et al. [55], using EPR spectroscopy.

**Figure 3:**
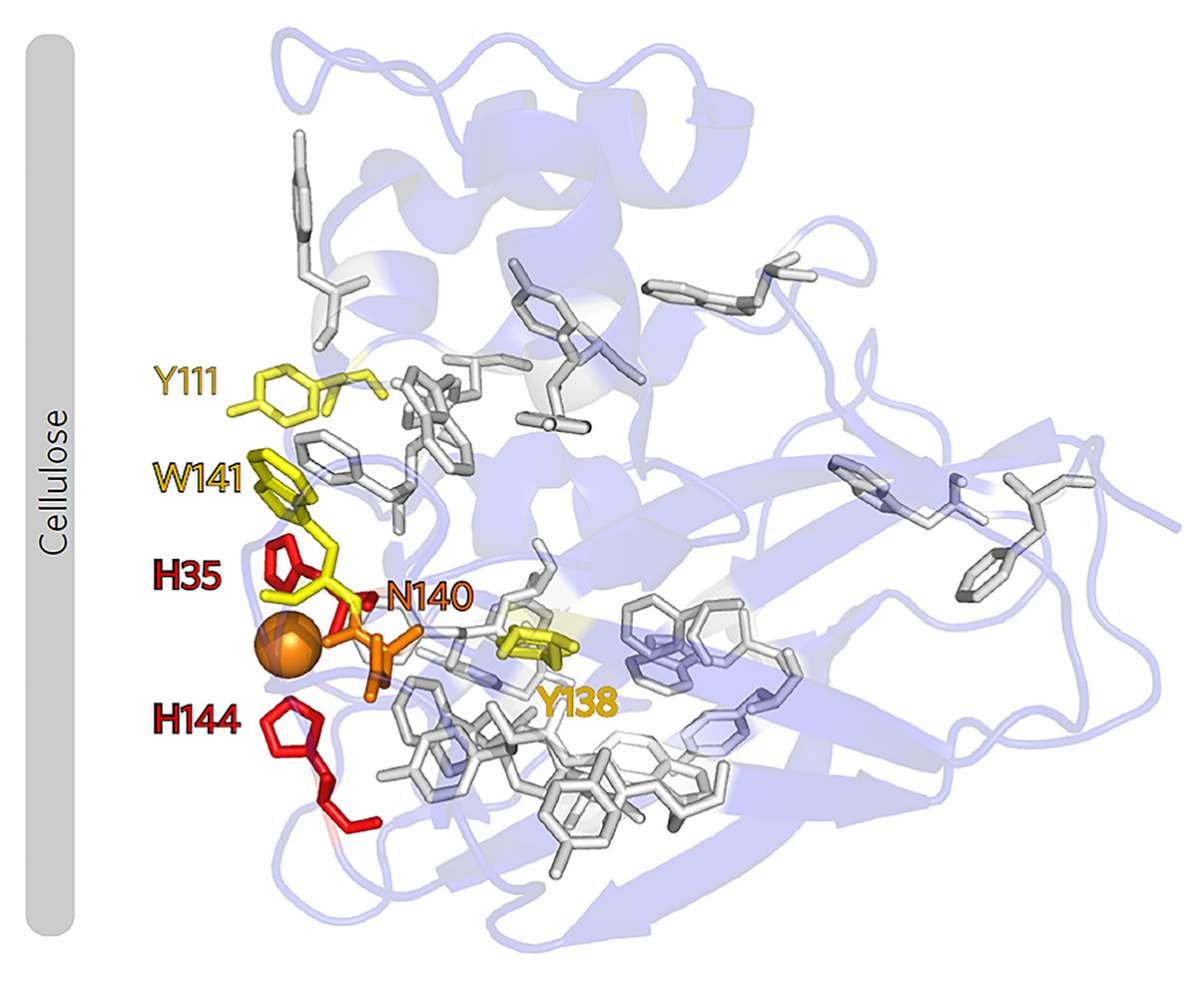
Oxidative damage in an AA10 LPMO Exposure of this LPMO to H_2_O_2_ in the absence of substrate showed that oxidative damage starts at the two catalytic histidines (shown as red sticks) and propagates to Tyr and Trp residues further away from the catalytic center [45]. The color code indicates the degree of oxidative damage, from high (red) to low (yellow). In the same study, it was also shown that productive binding to cellulose has a protective effect. Although the focus of this early work was not on protective mechanisms, these results clearly show that the radical generated in the catalytic center may propagate through the enzyme via aromatic residues. Figure taken from [45].

The notion that LPMO activity involves Cu(I)-catalyzed formation of a hydroxyl radical from H_2_O_2_ supports the original suggestion that LPMOs enable controlled, site-specific exploitation of the power of Fenton chemistry [44]. The ability to generate hydroxyl radicals is not without risks as evidenced by the autocatalytic inactivation of LPMOs through reactions with H_2_O_2_ in the absence of substrate ([45]; Figure 3; more below). Notably, for LPMOs studied so far, this reaction seems rather slow (∼10^3^ M^−1^ s^−1^) [30] compared with the productive reaction with substrate (∼10^6^ M^−1^ s^−1^) [30,52] (note that these rates may vary between LPMOs). This rate difference shows that the confinement of the LPMO-substrate complex provides the appropriate molecular environment to generate the hydroxyl radical and then use it in a productive manner. In a later study, Wang et al. referred to this as a ‘caged radical’ [29]. Such tight enzymatic control is truly remarkable given that such radical is highly reactive, with a half-live of about 1 ns in biological systems, and underscores the importance of the substrate in protecting the LPMO from autocatalytic inactivation.

While it is clear that LPMOs are efficient peroxygenases and while it is now well established that H_2_O_2_ will be formed *in situ* in LPMO reactions, one key remaining question is whether LPMOs catalyze monooxygenase reactions at all. There is no doubt that reduced LPMOs can activate molecular oxygen [41,66]. Furthermore, there are computational indications that substrate-binding stabilizes the Cu(II)-superoxide species [40], which is otherwise prone to dissociation [66]. Modeling studies suggest that a monooxygenase reaction is feasible (via a Cu(II)-oxyl), and mechanisms for the timely delivery of the electrons and protons needed to reach the Cu(II)-oxyl state have been proposed [43,69]. Yet, such delivery mechanisms remain to be experimentally validated. Interestingly, based on quantum mechanics/molecular mechanics (QM/MM) metadynamics simulations, Wang et al. [29] have proposed a monooxygenase reaction involving the formation of H_2_O_2_ (from O_2_) within the active site cavity formed by the LPMO-substrate complex on the productive catalytic pathway. This proposal has been used by some to claim that the peroxygenase reaction is some sort of ‘shunt’ for what essentially would be a monooxygenase reaction. While this proposed catalytic pathway cannot be excluded, it fails to explain several experimental observations (see above), including the ‘second electron conundrum’ and the well-documented inhibition of LPMO activity by H_2_O_2_-consuming enzymes. Also, we believe that the use of the term ‘shunt’ is misleading, since in the field where this term has originally been used to describe oxygen-dependent redox catalysis (P450 cytochromes), the shunt pathway refers to a ‘forced’, rather slow and inefficient pathway that requires exceedingly high amounts of H_2_O_2_ (several mM) to bypass the electron/proton delivery steps of the standard O_2_ pathway. For LPMOs, the situation is actually nearly the opposite, since recent kinetic data clearly show that the peroxygenase reaction is orders of magnitude faster than the monooxygenase reaction [47,52,54–56] and that stable peroxygenase reactions with total turnover numbers of tenths of thousands can occur at the low steady-state H_2_O_2_ concentrations (µM range) found in microbial ecosystems [59].

## Assessment of the LPMO literature in light of recent findings

Due to the many biotic and abiotic processes that may be going on in typical LPMO reactions, reliable kinetic characterization of LPMO catalysis is challenging, as discussed elsewhere [70,71]. Data on the catalytic action of LPMOs need to be analyzed with great care, for example due the possible occurrence of enzyme inactivation, and the occurrence of multiple H_2_O_2_-consuming and -producing (side-) reactions. It cannot be emphasized enough that many published LPMO reactions likely were limited by *in-situ* generation of H_2_O_2_ and not by, e.g. the concentration of active LPMO. Older LPMO studies with quantitative statements on LPMO activity may need some reassessment.

When reading the literature, one also has to realize that the use of H_2_O_2_ vs O_2_ as cosubstrate has been, and to some extent still is, controversial. The literature contains several claims that may have been colored by the ongoing debate and that perhaps need some critical reassessment:

- It has been claimed by some that autocatalytic inactivation of LPMOs primarily happens in H_2_O_2_-driven reactions and that this shows that the peroxygenase reaction is not natural or optimal, in contrast with the apparent monooxygenase reaction. There are no data in the literature supporting such claims. It is actually well documented that for natural H_2_O_2_-dependent enzymes, such as peroxidases and peroxygenases, high amounts of H_2_O_2_ lead to high enzyme rates but also to enzyme damage [45,47]. Such damage may not always be seen under ‘monooxygenase conditions,’ but this is simply due to the steady-state concentration of H_2_O_2_ being lower, as is the reaction rate.
- It has been claimed that the peroxygenase reaction is less specific [47]. However, we note that most published studies do not report differences between product profiles generated in apparent monooxygenase and peroxygenase reactions (e.g. [72]). Generally, one may see trace amounts of nonstandard products, e.g. in mass spectra, and the occurrence of such products varies between reactions. We would argue that oxidative damage to the catalytic center, which starts at the histidines [45], could lead to loss of specificity, for example due to a less well-controlled hydroxyl radical or minor changes in the enzyme-substrate interaction.

## Concluding remarks

Metal-containing redox enzymes are difficult to study due to the many possible off-pathway reactions, the susceptibility of the enzyme to oxidative damage, and challenges related to obtaining reliable kinetic data. The LPMOs are no exception and research on these enzymes has pointed at one challenge in particular, namely conclusive identification of the true oxygen cosubstrate of what could be a monooxygenase or a peroxygenase reaction. The quest to understand the nature of LPMO catalysis has led to a much-debated paradigm change, with LPMOs going from being considered as monooxygenases to being recognized as efficient peroxygenases that may possibly act as slow monooxygenases. While the peroxygenase reaction of LPMOs provided plausible answers to outstanding questions in the field (as discussed above), and seems ‘logical’ in the context of how fungal secretomes work and have access to various oxygen species [42,59], it was met by considerable skepticism. One (understandable) reason for such skepticism likely was that LPMOs are truly unique peroxygenases, with an at the time unprecedented active site architecture. Indeed, the large majority of known peroxygenases are heme enzymes [73] and, to the best of our knowledge, LPMOs are the only monocopper enzymes for which peroxygenase activity has been convincingly demonstrated. Of note, multiple studies have shown that LPMOs and heme-peroxygenases have similar catalytic efficiencies [52,54,56,58,61].

Interestingly, other types of enzymes have also been suggested to make use of Fenton-type O–O homolysis of H_2_O_2_ to catalyze oxidative transformations, such as some heme-peroxidases, some families of cytochrome P450 and the nonheme HppE [74]. In these cases, the HO^•^ species is either directly used as a substrate oxidant or, as in LPMO catalysis, used to generate the enzymatic active species (e.g. Compound I (Cpd I), Por^+•^Fe(IV)═O in P450s). HppE is a particularly interesting case since it was shown that this nonheme monoiron epoxidase involved in fosfomycin production, is not an oxidase (i.e. does not use O_2_) as previously thought, but a peroxidase, using H_2_O_2_ [75]. Clearly, identification of the oxygen cosubstrate is challenging and it is important to carefully consider the entire chain of reactive oxygen species when studying the enzymes discussed above. It cannot be excluded that additional ‘mistakes,’ similar to those made for LPMOs and HppE, will be discovered in the future. In this light, it is interesting to note that modeling studies suggest that a peroxygenase reaction is energetically feasible for particulate methane monooxygenase, which needs to overcome an even higher energy barrier than LPMOs [76].

Despite major advances achieved in the LPMO field over the past decade, several important questions remain. For example, the species likely abstracting the hydrogen from the substrate, the Cu(II)-oxyl, has not yet been observed experimentally. Turning to biology, questions remain as to the levels of O_2_ and H_2_O_2_ in biomass-degrading ecosystems and the actual levels of LPMO activity in such systems. Another interesting issue, of utmost importance for both understanding biological systems and industrial applications, concerns the stability of LPMOs under turnover conditions and the possible existence of protective mechanisms. The surfaces and cores of LPMOs contain aromatic amino acids that vary in number and location; it is quite conceivable that at least in some LPMOs, these residues may participate in so-called hole-hopping pathways [77] that help dissipating potentially damaging radicals from the catalytic center (Figure 3). Indeed, recent studies demonstrate the formation of aromatic radicals in LPMOs [55,56,78,79].

Perhaps, one of the most intriguing issues concerns the huge multiplicity of LPMOs in fungi [80] and the proven roles of LPMOs in microbial pathogenicity [11]. The true substrates of these many LPMOs remain unknown. While it is easy to envisage a need for powerful, Fenton-like chemistry for degradation of crystalline cellulose or complex copolymeric substructures in plant cell walls, it is not at all clear what type of recalcitrant substrate, if any, is being degraded/targeted by several LPMOs reported in the literature, such as CbpD, a virulence factor produced by *Pseudomonas aeruginosa* [16]. Further research is needed to reveal how Nature employs the power of site-specific, well-controlled Fenton-type chemistry to solve highly challenging catalytic tasks, some of which are well known, while others remain to be discovered.

## Summary

- LPMOs are abundant enzymes that play a key role in the enzymatic conversion of recalcitrant polysaccharides, such as cellulose and chitin, and that may have other functions related to cellular development and microbial virulence.
- Since their discovery, LPMOs have progressed from being considered slow monooxygenases to being considered efficient peroxygenases.
- These unique peroxygenases, with a single copper ion as the only cofactor, harness the power of Fenton-type chemistry, i.e. copper-catalyzed homolytic cleavage of hydrogen peroxide to generate a hydroxy radical, in a controlled, site-specific manner.
- Topics for further studies include the detection of the hydrogen-abstracting oxygen species, predicted to be a Cu-oxyl rather than the hydroxyl radical, the substrates of LPMOs involved in cellular function rather than biomass conversion, and the existence of hole-hopping mechanisms that protect LPMOs from autocatalytic inactivation.

## Abbreviations

AA: auxiliary activities
CBM33: a family 33 carbohydrate-binding module
EPR: electron paramagnetic resonance
GH61: a family 61 glycoside hydrolase
haa: hydrogen atom abstraction
LPMO: lytic polysaccharide monooxygenase
